# *Macrostomum lignano* Complements the Portfolio of Simple Animal Models Used for Marine Toxicological Studies

**DOI:** 10.3390/ijms252313092

**Published:** 2024-12-05

**Authors:** Yuanyuan Ma, Thomas Roeder

**Affiliations:** 1 Nantong Key Laboratory of Environmental Toxicology, Department of Occupational Medicine and Environmental Toxicology, School of Public Health, Nantong University, Nantong 226019, China; 2 Zoological Institute, Molecular Physiology, Kiel University, Olshausenstrasse 40, 24098 Kiel, Germany; 3 German Center for Lung Research (DZL), Airway Research Center North, 24098 Kiel, Germany

**Keywords:** *Macrostomum*, model organism, toxicology, marine pollution, waste, behavior, environmental pollution indicator

## Abstract

*Macrostomum lignano* is gaining increasing recognition as a model organism for toxicological studies in marine ecosystems and expands the range of simple animal models currently used. Water pollution caused by human activities not only endangers environmental integrity but also affects human health, underlining the need to monitor water pollution effectively. This review describes the distinctive characteristics of *M. lignano*, including its rapid reproductive cycle, increased sensitivity to environmental variability, and remarkable regenerative abilities. Over the last thirty years, *M. lignano* has been used in various research areas, particularly molecular biology and toxicology. This endeavor has benefited from significant advances in genome and transcriptome technologies. Recent investigations have revealed its sensitivity to various pollutants and highlighted its potential for assessing toxicological effects at the physiological and molecular levels. Furthermore, the ecological versatility and stable microbiome of *M. lignano* make it an exemplary model for research into pollutant interactions in marine ecosystems. Despite challenges associated with its complex genomic architecture, ongoing genomic efforts are promising to significantly enhance its utility in toxicological research. This review underscores the pivotal role of *M. lignano* in advancing environmental health studies and outlines future research directions to maximize its potential as a model organism.

## 1. Introduction

Water pollution caused by human activities such as using fertilizers, industrial mining, smelting, and the generation of municipal waste is causing severe environmental problems [1]. Globally, water pollution causes death and disease, and approximately 14,000 people die every day due to water pollution [2,3,4]. Some of these persistent pollutants can biomagnify and accumulate in the food chain [5]. Therefore, monitoring and assessing the effects of contaminants in the aquatic environment is crucial for protecting human health and the environment. In aquatic toxicology research, different model organisms are utilized due to their unique characteristics and advantages. For instance, the zebrafish (*Danio rerio*) is one of the most commonly employed aquatic model organisms, notable for its transparent embryos, short life cycle (approximately 3 months), and a rich array of genetic and molecular biology tools [6]. These features make it well-suited for studies in developmental biology, toxicology, and behavior [7,8,9,10]. The common carp (*Cyprinus carpio*) is widely distributed in freshwater ecosystems globally, offering high ecological representativeness and sharing numerous physiological and biochemical pathways with humans. This makes it an ideal model for investigating the long-term impacts of environmental pollutants on both ecosystems and human health [11,12]. Mollusks, such as *Mytilus edulis* and *Pecten maximus*, play crucial roles in aquatic ecosystems and exhibit high sensitivity to environmental changes, making them valuable bioindicators [13,14]. However, their genetic and molecular biology tools are relatively limited [15]. Algae are ubiquitous in various aquatic environments, characterized by rapid growth rates, which make them suitable for high-throughput screening and short-term experiments. They are particularly useful for studying the effects of pollutants on primary producers and for examining carbon cycling and energy flow [16] (Table 1).

This review focuses on the suitability of *Macrostomum lignano* as the model organism for toxicological studies. Parlak (2024) [17] underscores that *M. lignano* has emerged as a promising model for aquatic toxicology research. *M. lignano*, a member of the order Macrostomorpha, is a basal member of the meiobenthic flatworms. Since its introduction into laboratories almost 30 years ago [18], *M. lignano* has been used as a model organism for research in various fields, including stem cell biology, developmental biology, regeneration, aging, sex determination, sperm competition, bioadhesion, and microbiome studies [19,20,21,22,23,24,25,26]. In the past decade, significant progress has been made in the development of molecular tools for this organism, including the generation of transcriptome and genome assemblies and improvements in transgenic methods [27,28,29]. Studies have shown that *M. lignano* exhibits increased sensitivity to fluctuations in environmental compounds. In particular, exposure to fluctuations in salinity and heavy metal ions can induce changes in relevant life history traits and physiological and behavioral responses [30,31]. This review comprehensively describes the inherent biological properties of *M. lignano* that position it as a valuable experimental model. It also explores recent advances in the development of molecular tools and resources that have further expanded the research potential of this model organism, particularly in the field of toxicology. Through this article, we aim to elucidate the fundamental biological characteristics that make *M. lignano* an advantageous experimental model, highlight recent advances in the development of molecular tools, and emphasize the promising role of this model organism in advancing toxicology-related research.

**Table 1:** Comparative analysis of model organisms in environmental toxicology.

Category	Common Types	Characteristics	Advantages	Limitations	Applications	Specific Research	References
Vertebrata	*Danio rerio*	Transparent embryos; short life cycle; mature genetic and molecular biology tools.	Abundant genetic and molecular tools; high ecological representativeness; multifunctionality.	High cost; long experimental cycle.	Evaluating the effects of chemical substances on development and growth, including neurotoxicity, cardiotoxicity, endocrine disruption, etc.	Testing the effects of pesticides, heavy metals, and endocrine disruptors on embryonic development, etc.	[7,8,9,10]
	*Cyprinus carpio*	Widely distributed; large organisms; relatively mature genetic tools.	Ecological representativeness; physiological and biochemical similarity to humans.	High maintenance cost; long experimental cycle.	Widely used to evaluate the impact of water pollution.	Monitoring the toxic effects of heavy metals, pesticides, and other chemical substances.	[11,12]
Mollusca	*Mytilus edulis*; Pecten maximus	Diverse and representative ecological roles; sensitive to environmental changes.	Ecological representativeness; biological monitoring.	Limited genetic tools; relatively complex experimental design and operation.	Evaluate the impacts of heavy metals, organic pollutants, and pharmaceuticals on aquatic ecosystems.	Measuring the bioaccumulation rates and physiological responses of mollusks can assess the long-term impacts of pollutants.	[13,14,15]
Platyhelminthes	*Macrostomum lignano*	Small and transparent; short life cycle; strong regenerative capacity; well-established genetic manipulation techniques; easy to culture.	Easy high-resolution imaging; rapid experimental turnaround; regenerative research.	Weak ecological representativeness; fewer tools and resources.	Evaluate the impacts of heavy metals, organic pollutants, and pharmaceuticals on aquatic ecosystems.	Toxicity testing, regenerative capacity studies, and assessing behavioral impacts (such as movement, feeding, etc.), including evaluation of neurotoxicity, reproductive toxicity etc.	[26,30]
Algae	*Chlamydomonas reinhardtii;* Synechocystis sp.; Navicula pelliculosa	Widely distributed; rapid growth; photosynthesis.	Ecological representativeness; rapid experimental turnaround.	Complexity; limited genetic tools.	Evaluate the impact of chemical substances on primary productivity, including photosynthesis, cell growth, and reproductive capacity.	Measuring the growth rate, chlorophyll content, and photosynthetic efficiency of algae can assess the short-term and long-term impacts of pollutants on algae.	[16,32,33,34]

## 2. Biological Traits and Laboratory Cultivation of *M. lignano*

*Macrostomum lignano*, a tiny transparent marine flatworm measuring about 1.5 mm in length [22], showcases a slender, elongated body with a distinct brown coloration. This organism’s life cycle demonstrates significant reproductive flexibility and is capable of both sexual and asexual reproduction through fission and regeneration processes [35]. Moreover, *M. lignano* stands out for its brief generation time, rapid embryonic development, and high reproductive rates [36]. The cultivation of *M. lignano* in laboratory conditions is straightforward, given the vigorous growth of worms in Petri dishes containing nutrient-enriched artificial seawater with a salinity of 32 ‰ [37]. They are typically maintained at 20 °C under a light/dark cycle of either 13 h light and 11 h dark or the optimal selected conditions based on the experimental conditions [21]. These flatworms are given the diatom *Nitzschia curvilineata* as their food source, and they are cultivated under similar conditions [38]. *M. lignano*, an obligatory self-incompatible hermaphrodite that reproduces exclusively sexually, enables the rapid establishment of large-scale cultures [19], with adult worms laying around one egg per day at 20 °C. The embryonic development spans 5 days, contributing to a total generation time of approximately 18 days [22]. In contrast to planarians, *M. lignano* exhibits quartet spiral embryonic cleavages in the early stages and undergoes “inverse epiboly” in later development phases [39]. The eggs of *M. lignano* display entolecithal (archoophoran) development, characterized by a large single cell size of 100 μm, making them suitable for microinjections [40].

## 3. The Habitat of *Macrostomum lignano*—Unique for Marine Models

*M. lignano* is predominantly found in intertidal habitats [18], such as sandy beaches and mudflats, where it thrives in shallow marine waters with a high concentration of inorganic and organic matter. The discharge from sewage systems has the potential to increase pollutant levels rapidly, while alterations in the natural surroundings can result in fluctuations in toxic substance concentrations within intertidal zones [31]. Environmental changes, such as the combination of tidal effects with high temperatures and significant evaporation commonly observed during the summer months [30], can lead to a swift rise in water pollutant levels. *M. lignano* showcases impressive evolutionary adaptability through its opportunistic feeding behavior on small invertebrates and organic debris found in microhabitats such as bivalve shells and algal mats. These microhabitats not only serve as shelter but also as sources of food. This marine habitat is unique as it combines this enormous degree of changes in major characteristics that can occur at different time scales with being the marine habitat most affected by human activities.

## 4. Experimental Tractability

### Growth and Reproductive Capacity

The single-cell fertilized egg at the tail of the transparent worm body is clearly visible during the five days of development. The single cell has a relatively large size of 100 μm [40]. From this fertilized egg, a juvenile hatches through a predetermined opening in the eggshell and undergoes direct maturation into a fully developed organism around two weeks [26,41]. Statistical analysis of egg and hatching rates can be conducted through microscopic examination, but they are also open to automation. The changed growth rate acts as a critical marker, enabling the evaluation of sensitivity to substance toxicity. Any decrease or halt in the rate of proliferation provides clear evidence of the consequences on biological populations, emphasizing the effects on reparative mechanisms.

## 5. Volume Measurements

Changes in worms’ volume indicate modifications in their body structure and indirectly signify fluctuations in the biological osmotic pressure in *M. lignano*. Worms can be immobilized using 7.14% MgCl_2_ as an anesthetic to prevent stress-induced muscle contractions [42]. The slender body structure of the worms not only enables the straightforward observation of morphological changes, even under low-magnification stereoscopes, but significantly enhances the efficacy of confocal imaging procedures.

## 6. Physical Activities

Changes in behavior can serve as indicators of the effects of pollutants on the functions of the biological nervous system and sensory organs, providing a crucial basis for assessing pollutant toxicity. Schärer et al. (2004) [43] introduced an innovative observation chamber for studying these effects, where worms can freely move within a droplet of f/2 medium sandwiched between two microscope slides. This setup allows the worms to be observed continuously for up to 10 days without additional food. Microscopic imaging enables effective monitoring of individual movements. Deep red staining is employed to minimize the impact of laser effects on the worms and enhance recording quality, improving the contrast between the worms and the background. Recording duration and frequency are adjusted according to the worms’ activity levels, ensuring that animals with higher activity levels are monitored more frequently. This approach results in more images captured within the same time frame. Image processing using ImageJ version 1.54 software, followed by analysis in Imaris with the “Imaris Track” module by BitPlane, facilitates the extraction of parameters such as distance, average, or maximum values [30].

## 7. Respiration Rate Measurements

Respiration rate measurements provide valuable insights into organisms’ metabolic status and overall health. A fiber-optic oxygen meter (Oxy-4) was employed with non-invasive oxygen sensors to conduct these measurements (SP-PSt3-NAU-D5-YOP, Precision Sensing, Regensburg, Germany). Each measurement session involved approximately 20 animals to minimize data fluctuations [44]. The respiration rates were quantified and presented as individual oxygen consumption per unit time. The critical oxygen partial pressure (Pc) was determined using an equation derived from the method proposed by Tang (1933) [45] and further developed by Duggleby (1984) [46].

## 8. Total Feces Output

Variations in fecal characteristics, including color, texture, odor, and defecation speed, are essential indicators of intestinal function, reflecting gastrointestinal health, nutrient absorption, gut microbiota equilibrium, and overall motility. Monitoring these features provides critical insights for assessing and managing various gastrointestinal disorders. *M. lignano’s* fecal samples can be quantified using well plates with daily medium changes [26]. Selecting suitable time points for image capture and quantitative analysis is based on experimental requirements. This method ensures comprehensive data collection and analysis to assess the influence of different culture conditions on fecal characteristics.

## 9. Live-Imaging Techniques

Live-imaging techniques hold great promise for real-time monitoring of complex biological processes in a large population of small animals. The transparent nature of the meiofauna organism *M. lignano* makes it highly suitable for toxicological studies, especially those focusing on whole-animal investigations to understand toxic effects at the molecular and cellular levels. Mitochondrial density, energy status, and reactive oxygen species (ROS) production can be quantified using staining dyes and probes on anesthetized worms. *M. lignano’s* small size and transparency facilitate the application of standard staining and immunohistochemistry techniques without dissection. This can be supported by phospho-H3 (pH3) labeling for observing mitotic cells and ethidium homodimer III staining for identifying dead cells [31]. Additionally, cell proliferation can be evaluated using BrdU staining techniques [47].

## 10. Assessing Tissue Healing and Regeneration

In toxicology research, assessing tissue healing and regeneration is a pivotal indicator for gauging the recovery and rejuvenation of injured tissues. *M. lignano*, distinguished by its robust regenerative prowess, boasts a substantial reservoir of stem cells known as neoblasts. This distinct characteristic sets *M. lignano* apart from other model organisms. Notably, experimental models for amputation and subsequent repair timelines have been successfully established across various anatomical regions of *M. lignano*, with a typical recovery period ranging from 1 to 3 weeks [41].

## 11. RNA Interference and In Situ Hybridization

RNA interference (RNAi) and in situ hybridization (ISH) are pivotal techniques for investigating gene function and understanding the spatiotemporal expression patterns of genes. Mouton et al. (2024) [42] optimized RNAi protocols, demonstrating that a single soaking in artificial seawater with double-stranded RNA (dsRNA)—either produced in bacteria or synthesized in vitro—can effectively induce robust gene knockdown for several days, even in the absence of diatoms.

In addition, Lengerer et al. (2014) [24] developed a comprehensive Whole Mount In Situ Hybridization (WM-ISH) method. This approach involves several key steps: designing primer sequences that incorporate a T7 promoter, amplifying the template DNA via PCR, and synthesizing single-stranded RNA probes labeled with digoxigenin using T7 polymerase and a DIG labeling mix. Subsequent steps include diluting the anti-digoxigenin-AP Fab, visualizing the signal at 37 °C with NBT/BCIP, and mounting specimens in Mowiol^®^ 4–88 for microscopy visualization.

Together, these methodologies enhance our capability to analyze gene expression and function, providing valuable insights into the underlying biological processes.

## 12. Genome, Transcriptome, and Genome Browser Analysis

*M. lignano*’s genome exhibits a remarkable level of manipulability, as demonstrated by the successful establishment of transgenesis techniques utilizing its easily manipulable single-cell zygotes derived from abundant egg stores [48]. Currently, it stands as the only flatworm with the capability for stable transgenesis [49]. The annotated genome assembly, Mlig_3_7_DV1 [29], is readily accessible via GenBank (accession no. NIVC00000000.1) and the Macrostomum genome resources website (http://www.macgenome.org (accessed on 15 October 2024)). Researchers can further explore this genome using the UCSC genome browser interface (Table 2).

Through the application of gene editing technologies, scientists can precisely alter gene expression related to pollutant exposure in *M. lignano*’s genome, thereby revealing the functions and roles of these genes within the organism. By fluorescently tagging specific genes, an environmental surveillance animal model can be established, providing a valuable tool for toxicological investigations and offering deeper insights into how environmental pollutants impact organisms.

**Table 2:** Public web-based resources for flatworm *Macrostomum lignano* genomics.

Title/URL	Notes	Reference
NCBI Genome Resources https://www.ncbi.nlm.nih.gov/datasets/genome/GCA_002269645.1/ (accessed on 20 November 2024)	version Mlig_3_7_DV1	[29]
Flatworms and Acoels Genome Browser http://gb.macgenome.org (accessed on 20 November 2024)	version Mlig_4_5; Mlig_3_7	[40]
*Macrostomum lignano* genome resources website http://www.macgenome.org (accessed on 20 November 2024)	version Mlig_3_7; Mlig_RNA_3_7_DV1_v3; Mlig_RNA_3_7_DV1_v1	[40]
WormBase ParaSite https://parasite.wormbase.org/Macrostomum_lignano_prjna284736/Info/Index/ (accessed on 20 November 2024)	version Mlig_3_7_DV1	[27]

## 13. The Versatility and Challenges of *Macrostomum lignano* in Toxicology Research

*M. lignano*, though still emerging as a model organism in toxicological research, has rapidly gained recognition for its utility in toxicity testing and chemical exposure studies. Its notable sensitivity to diverse environmental stressors, coupled with well-defined biological responses to various toxicants, positions *M. lignano* as a valuable tool for researchers in this field. Studies have utilized this organism to evaluate acute and chronic toxic effects of contaminants, including inorganic and organic compounds, through careful monitoring of survival rates and physiological parameters following exposure [31,50]. Specifically, environmental concentrations of copper and cadmium are reported to range from 150 to 375 μM and 6 to 2500 μM, respectively [51,52]. In experiments, *M. lignano* exhibited lethality at copper concentrations above 10 μM and cadmium concentrations exceeding 330 μM [31]. Furthermore, *M. lignano* demonstrates a significant sensitivity advantage over *Cyprinodon variegatus* in detecting the transformation products of TNT, namely 2-aminodinitrotoluene (2-ADNT) and 2,4-diaminonitrotoluene (2,4-DANT), with lethal doses reduced by approximately 40%. Remarkably, behavioral alterations in feeding were observed at a concentration as low as 0.033 mg/L [50,53].

Overload of environmental substances, even salinity, leads to a range of physiological effects in *M. lignano*, including elevated cell death events, alterations in mitochondrial biology, and reduced physical activity. At the molecular level, gene expression analyses reveal substantial alterations in several genes associated with xenobiotic metabolism in response to these interventions. Among these, glutathione S-transferase pi-1 emerges as a critical anti-redox gene that plays a significant role in the organism’s response to oxidative stress and xenobiotic exposure [30,31]. These findings underscore the complex interplay between environmental stressors and the physiological and molecular responses of *M. lignano*, highlighting its potential as a model for understanding toxicological mechanisms.

Cell proliferation is a fundamental endpoint for assessing the carcinogenic potential of chemical substances. *M. lignano*, characterized by its high proportion of stem cells—approximately 6.5% of its total 25,000 cells—distinguishes itself from other model organisms such as zebrafish and algae [54]. Notably, about 27% of these somatic neoblasts are in the S-phase, indicating significant cellular activity [54,55,56,57]. The organism’s epidermis exemplifies this regenerative capacity, with around one-third of its cells renewed within a two-week cycle [55]. This remarkable regenerative ability offers valuable opportunities for substance safety testing and the in vivo evaluation of dynamic changes in adult pluripotent stem cells. For example, Pfister et al. (2007) [47] investigated the effects of hydroxyurea (HU) treatment and irradiation on gene expression in *M. lignano*, focusing on two critical stem cell- and germ cell-related genes, *macvasa* and *macpiwi*. Further reinforcing the utility of this model, Willems et al. (2015) [58] assessed four known carcinogens, two that interact with DNA and two that do not, demonstrating the viability of lower organisms as alternatives to higher animals for carcinogenicity testing. Moreover, the microbiome of *M. lignano* presents a promising model for toxicological research, as it demonstrates a stable host-microbiota relationship across different developmental stages, each characterized by a distinct microbiota. Notably, the microbiota exhibits circadian rhythmicity, influencing both the overall bacterial load and the behavior of specific taxa [26]. Studies with gnotobiotic worms have demonstrated the crucial role of the microbiota, showing that while no fitness advantages emerge when food is plentiful, worms harboring a microbiota exhibit significantly improved fitness under limited food conditions. In experiments assessing the impact of pesticides and herbicides, including deltamethrin and atrazine, significant alterations in the microbial community were observed (unpublished data). These findings underscore the potential of *M. lignano*’s microbiome as a model for further exploration within the realms of aquatic toxicology and environmental health.

In addition, *M. lignano* possesses distinct advantages over related parasitic flatworms, such as a simplified life cycle, low cultivation costs, and specialized anatomical and physiological adaptations for parasitism. These characteristics make *M. lignano* an ideal model for identifying and studying homologous genes [49]. Despite its immense potential as a model organism for toxicology studies, several challenges and limitations must be overcome to enhance the application of *M. lignano* in research. Notably, *M. lignano* is the only species within its group that currently has a published genome assembly. However, its utility is hindered by the fact that it is a hidden polyploid, having recently undergone significant genomic events such as whole genome duplication and chromosome fusion [27]. This intricate genome architecture poses considerable obstacles to the application of many contemporary genetic tools and methodologies, which often rely on simpler, more stable genomes for accurate analysis.

To address these challenges, there is an urgent need for additional genomic resources within this genus. Recent efforts, such as those by Wiberg et al. (2023) [59], have aimed to mitigate these issues through comparative genomic research. They successfully sequenced the genomes of two sister species, *Macrostomum cliftonense* and *Macrostomum hystrix*, which may provide insights that help resolve the assembly ambiguities associated with *M. lignano*. By enhancing understanding of the genetic underpinnings of these closely related species, researchers may be better equipped to tackle the complexities of *M. lignano*’s genome. This, in turn, could bolster its position as a valuable model for toxicological research, facilitating advancements in comprehension of chemical safety and environmental health. Overall, while the path forward may be fraught with challenges, ongoing genomic initiatives hold promise for unlocking the full research potential of *M. lignano* and its relatives.

Furthermore, gaining a comprehensive understanding of the ecological relevance of *M. lignano* within broader marine ecosystems is vital for its effective application in environmental toxicology studies. Investigating the interactions between *M. lignano* and other marine organisms, as well as evaluating its sensitivity to various environmental stressors, is essential for assessing its ecological significance and its potential as a model organism. Despite the challenges that lie ahead, ongoing genomic initiatives and ecological research hold great promise for unlocking the full research potential of *M. lignano* and its relatives. Such advancements likely contributed significantly to the advancement of knowledge in the field of toxicology and environmental sciences.

In environmental science and toxicology research, different organisms are used for various experimental purposes, involving several key concepts. Laboratory species, such as zebrafish (*Danio rerio*) and fruit flies (*Drosophila melanogaster*), are organisms that have been raised and studied under controlled laboratory conditions for extended periods, possessing stable genetic backgrounds and well-characterized biological traits [60,61]. Experimental species are chosen based on specific research objectives and can be either laboratory species or wild-collected species [62]. Test organisms, such as water fleas (*Daphnia magna*) and algae (*Chlamydomonas reinhardtii*), are used to evaluate the toxicity and biological effects of chemical substances [63,64]. Bioassays are experimental methods that utilize living organisms to detect the biological activity and toxicity of chemicals or other factors [65]. Biomarkers, such as alanine aminotransferase (ALT) in serum, indicate specific molecular or physiological changes in organisms exposed to environmental stress or disease [66]. Bioindicators, like mosses and aquatic insects, reflect environmental quality or ecological conditions [67]. Sentinel species, such as seabirds and benthic invertebrates, are used to monitor and warn of potential environmental issues [68]. While these terms may overlap in some aspects, they each play unique roles in environmental science and toxicology research.

Depending on the research objective, *M*. *lignano* can serve as a laboratory species, experimental species, test organism, bioindicator, and sentinel species, as well as perform bioassays. The glutathione S-transferase (GST) within its body can act as a biomarker for toxic stress. This versatility makes *M*. *lignano* a highly valuable model organism, particularly in studying biological responses and mechanisms under complex environmental conditions.

In conclusion, *M. lignano* has significant potential as a model organism in toxicology studies, providing unique advantages for studying the impacts of toxicants on biological systems. Overcoming the mentioned challenges and limitations and exploring the suggested research areas will allow *M. lignano* to significantly contribute to the advancement of knowledge in the field of environmental toxicology and support human and environmental health.
